# The effect of an adjunct psychosomatic intervention on perceived pain in patients with musculoskeletal disorders: protocol for a randomized controlled trial

**DOI:** 10.1186/s13063-026-09593-8

**Published:** 2026-03-05

**Authors:** Nike Walter, Melvin Mohokum, Thomas Loew, Christian Heiss, Markus Rupp

**Affiliations:** 1 Department for Psychosomatic Medicine, University Hospital Regensburg, Regensburg, Germany; 2 Faculty Health, Medical and Life Sciences, University Furtwangen, Furtwangen, Germany; 3 Department of Trauma, Hand and Reconstructive Surgery, University Hospital Giessen, Giessen, Germany

**Keywords:** Musculoskeletal disorders, Chronic pain, Psychosomatic therapy, Guided meditation, Aromatherapy, Stress, Quality of life

## Abstract

**Background:**

Musculoskeletal disorders, including chronic back pain, osteoarthritis, rheumatoid arthritis, and fibromyalgia, are leading causes of chronic pain and reduced quality of life. Standard treatment approaches often focus on physical symptoms, while psychosomatic factors are sometimes overlooked. This study aims to evaluate the effectiveness of adjunct psychosomatic interventions—guided meditation and aromatherapy—on pain perception, stress levels, and quality of life in patients with musculoskeletal disorders.

**Methods:**

This is a three-arm, prospective, randomized controlled trial conducted in Germany. A total of 90 participants aged 18 to 90 with chronic musculoskeletal disorders will be randomized into one of three groups: (1) guided meditation, (2) aromatherapy, or (3) control receiving standard care. The intervention groups will receive either body-scan meditation (23 min, twice weekly) or aromatherapy (5–10 min daily) for one week. The primary outcomes include pain intensity (measured using the Numerical Rating Scale), perceived stress (measured using the Perceived Stress Questionnaire), and quality of life (measured using the EQ-5D). Secondary outcomes will include pain medication consumption. Follow-up assessments will be conducted at 3-, 6-, and 12-month post-intervention. Data will be analyzed using an intention-to-treat approach with ANCOVA for primary endpoints.

**Discussion:**

This trial will provide valuable insights into the effectiveness of psychosomatic interventions as adjunct therapies for managing chronic pain and stress in musculoskeletal disorders. If successful, these interventions could be implemented as cost-effective and non-invasive strategies to improve quality of life and pain management for patients with musculoskeletal conditions. The findings may also inform future studies on integrative treatment approaches for chronic pain conditions.

**Trial registration:**

The trial is registered at the German Clinical Trials Register (https://www.drks.de), registration number DRKS00034506.

## Introduction

### Background and rationale {6a}

Musculoskeletal disorders (MSDs) are among the most prevalent chronic conditions globally, significantly impacting pain, stress levels, and quality of life [1, 2]. Conditions such as chronic back pain, osteoarthritis, rheumatoid arthritis, and fibromyalgia are common MSDs that frequently lead to persistent discomfort and functional limitations. Despite advances in conventional medical treatments, many patients with these conditions continue to experience considerable pain and reduced quality of life, prompting a search for additional therapeutic options [3, 4].

Integrative therapies, including psychosomatic approaches, have gained attention as potential adjuncts to conventional treatments for managing chronic pain and stress. Psychosomatic therapies often encompass techniques aimed at enhancing the mind-body connection, which may offer benefits beyond those achieved through standard medical care alone. Interventions such as guided meditation and aromatherapy are designed to promote relaxation, reduce stress, and potentially mitigate pain through non-pharmacological means [5].

Guided meditation, for instance, is a practice that involves directing the participant’s attention to various parts of the body, fostering a state of mindful awareness and relaxation. This approach has shown promise in improving pain management and emotional well-being in various patient populations [6, 7]. Similarly, aromatherapy, which utilizes the inhalation of essential oils, is believed to exert calming effects on the nervous system and could serve as a complementary method for pain and stress relief [8, 9].

This study aims to explore the effects of these complementary psychosomatic therapies on pain, stress, and quality of life in patients with musculoskeletal disorders. By conducting a prospective, three-arm, randomized controlled trial, we will evaluate the effectiveness of guided meditation and aromatherapy compared to standard care. This research seeks to determine whether these integrative approaches can provide meaningful benefits to patients suffering from chronic musculoskeletal conditions. Thus, it is hypothesized that providing patients with easy-to-implement methods for self-regulation, stress management, and anxiety reduction will result in a clinically relevant increase in quality of life scores, reduced pain levels, and fewer limitations in performing daily tasks.

Despite increasing interest in integrative and psychosomatic approaches, several important research gaps remain. Current standard care for musculoskeletal disorders primarily focuses on pharmacological treatment, physiotherapy, and surgical approaches. While effective for many patients, these strategies often insufficiently address the psychological and stress-related contributors to chronic pain. Furthermore, non-pharmacological interventions such as meditation and aromatherapy are widely used in clinical practice but lack standardized implementation protocols and high-quality randomized controlled trials specifically targeting heterogeneous musculoskeletal populations. Existing studies are frequently limited by small sample sizes, short follow-up periods, and single-intervention designs [10, 11]. Therefore, a rigorously designed randomized controlled trial evaluating standardized psychosomatic adjunct therapies is needed to address this gap.

### Objectives {7}

The objective of this trial is to examine the effect of adjunct psychosomatic therapies on pain perception, stress, and quality of life in patients with (i) lower back pain, (ii) osteoarthritis, (iii) rheumatoid arthritis, and (iv) fibromyalgia.

Specifically, we aim to:

- Evaluate the reduction in perceived pain using the Numerical Rating Scale (NRS).
- Assess changes in stress levels using the Perceived Stress Questionnaire (PSQ).
- Examine improvements in quality of life with the EQ-5D questionnaire.

## Methods: participants, interventions, and outcomes

### Trial design {8}

**Fig. 1:**
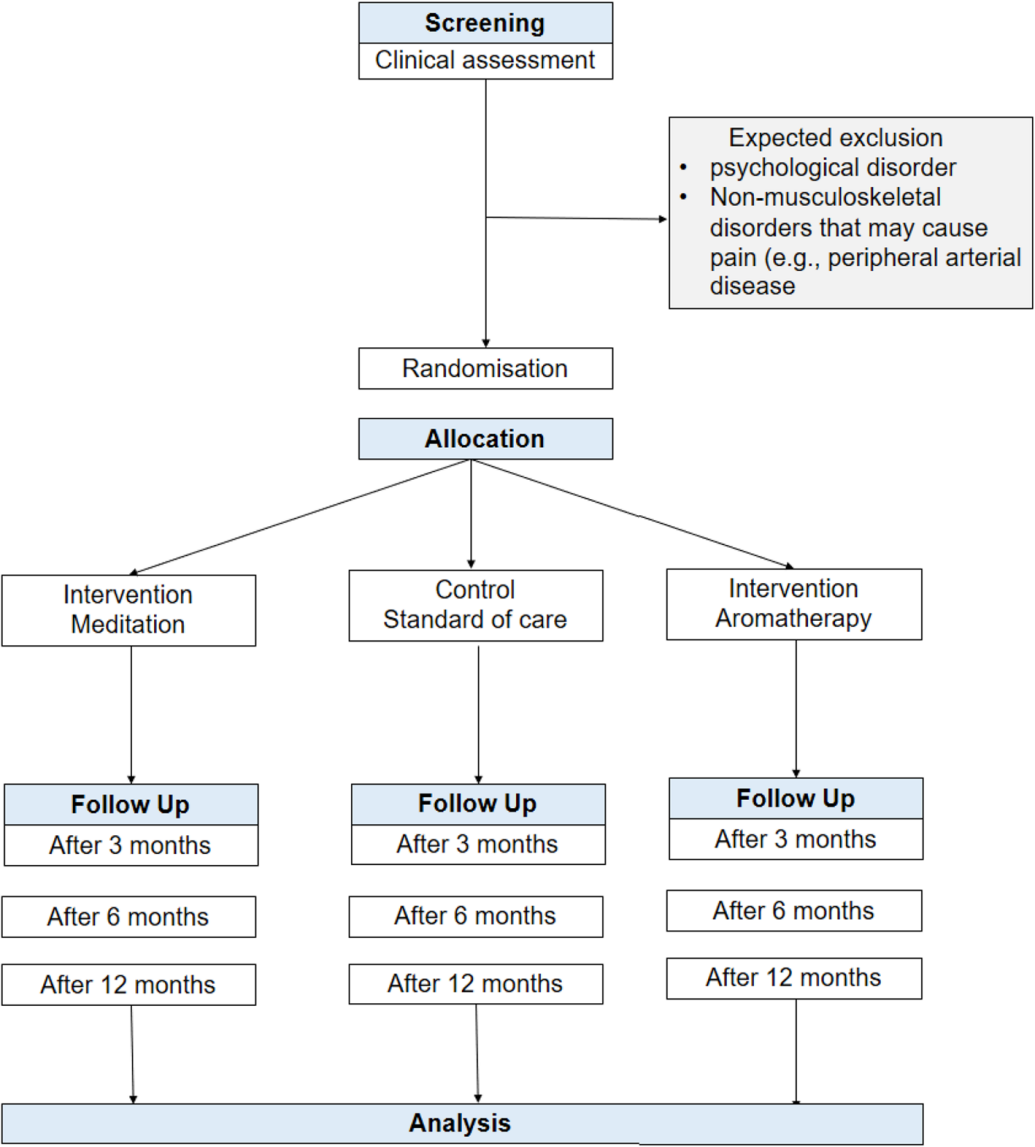
Schematic overview of the trial flow

This is a prospective, parallel three-arm randomized controlled trial (Fig. 1).

### Study setting {9}

The study will be carried out in four physiotherapy practices in Germany (REHA SÜD 79115 Freiburg, TheraVita GmbH 79111 Freiburg, Physiotherapie Gundelfingen GbR 79194 Gundelfingen, Körperwer 79098 Freiburg).

### Eligibility criteria {10}

#### Screening and diagnosis

Eligible participants will be screened using medical records, clinical examination, and confirmation of diagnosis by the treating physician.

### Diagnostic criteria

- Chronic lower back pain: pain ≥ 3 months confirmed by clinical assessment and imaging if available
- Osteoarthritis: diagnosis according to ACR criteria and/or radiographic evidence [12]
- Rheumatoid arthritis: diagnosis according to ACR/EULAR classification criteria [13]
- Fibromyalgia: diagnosis according to ACR 2016 criteria [14]

#### Inclusion criteria

- Age ≥ 18 years
- Confirmed diagnosis of one of the above musculoskeletal disorders
- Chronic pain duration ≥ 3 months
- Stable medical treatment for ≥ 4 weeks
- Ability to provide informed consent

#### Exclusion criteria

- Severe psychiatric disorders (psychosis, severe depression requiring hospitalization)
- Severe neurological disease
- Pregnancy or breastfeeding
- Participation in another clinical trial
- Current use of structured mind–body interventions (yoga, meditation programs, relaxation therapy)

#### Washout requirements

- NSAIDs: minimum 7-day washout before baseline assessment
- Complementary therapies (e.g., yoga, meditation, relaxation programs): discontinued ≥ 2 weeks before enrollment

### Drop-outs

Participants who withdraw consent or are lost to follow-up will be documented and included in intention-to-treat analyses.

### Who will take informed consent? {26a}

Participants will be approached in the physical therapy practice and given a verbal explanation of the study by a study researcher. A written participant information and consent form will be provided. Participants will be informed that their decision whether or not to participate in the study will not impact their access to routine care and that they can discontinue the participation in the study at any time. Participants will be given the opportunity to read, discuss, and ask questions. Those willing to participate will sign the consent form.

### Patient and public involvement

Patient representatives will be recruited through local patient advocacy groups and outpatient clinics using purposive sampling to ensure representation of the major diagnostic groups included in the trial. Patient representatives will be involved in the sense of participatory research [15, 16] in the conceptualisation of the study. These will participate during the whole research process. Further, focus group discussions will be held twice at University Hospital Regensburg, first to present the research strategy and preliminary results as well as to evaluate whether additional endpoints are of interest from the patient’s perspective. Second, it will be evaluated how patient-oriented, complementary treatment concepts can be established in daily clinical practice and medical aftercare. Regular meetings with the project partners will take place every 3 months.

### Additional consent provisions for collection and use of participant data and biological specimens {26b}

Additional written consent will be obtained for the collection and storage of blood samples for biomarker analysis (serum cortisol, C-reactive protein, and interleukin-6). Samples will be pseudonymized and stored according to institutional biobank regulations.

### Interventions

#### Explanation for the choice of comparators {6b}

In this study, two adjunct psychosomatic interventions—guided meditation and aromatherapy—will be compared to a control group receiving standard care as per current clinical guidelines. The choice of these comparators is based on the following rationale:

Guided meditation: This intervention involves a body-scan meditation that directs the participant’s focus on various body regions to promote mindfulness and relaxation. Guided meditation has been selected as it is a non-invasive technique widely used in psychosomatic medicine to reduce stress, alleviate pain, and enhance overall well-being [17–19]. The 23-min body-scan meditation, conducted twice weekly for 4 weeks, will be compared to standard care to evaluate its specific effects on pain perception, stress, and quality of life in patients with musculoskeletal disorders. The meditation protocol is based on standardized body-scan techniques derived from Mindfulness-Based Stress Reduction (MBSR).

Aromatherapy: Aromatherapy using fragrance sticks provides a simple, accessible intervention that could influence pain perception and stress reduction. It has been shown in some studies to have calming effects and improve emotional well-being, making it a suitable adjunct treatment [20, 21]. Participants will be instructed to use the fragrance sticks for 5–10 min daily for 1 week. The effects of this intervention will be compared to standard care to assess whether it offers additional benefits in managing chronic pain and stress. The fragrance sticks contain diluted essential oils compliant with EU cosmetic safety regulations. Participants will be screened for allergies and instructed to discontinue use in case of adverse reactions.

Control group (standard care): The control group will receive standard treatment based on clinical guidelines for managing musculoskeletal disorders. This includes routine medical care, physical therapy, and pain management protocols as applicable. The inclusion of a standard care group allows us to evaluate the added value of the psychosomatic interventions (meditation and aromatherapy) beyond conventional treatments. Standard care will follow national clinical guidelines and will be documented using a standardized checklist to ensure comparable treatment across centers.

### Intervention description {11a}

#### Group 1: Guided meditation

Participants will engage in a guided body-scan meditation, available through an online platform (https://www.tk.de/techniker/gesundheit-foerdern/stress-entspannung/aktiv-entspannen/body-scan-download-2007110, accessed 17.02.26) or via USB sticks/CDs. The meditation directs the participant’s attention to different body regions to raise awareness of bodily sensations and promote relaxation. Sessions will be conducted twice weekly for 23 min over 4 weeks.

#### Group 2: Aromatherapy

Participants will receive fragrance sticks for daily use (5–10 min per day). The participants will be instructed to use the sticks and provided with educational material on the therapeutic benefits of aromatherapy.

#### Group 3: Control (standard of care)

The control group will receive standard care as per existing clinical guidelines.

### Criteria for discontinuing or modifying allocated interventions {11b}

- for the individual patients: As guaranteed in the patient information sheet previously to study inclusion, the individual patient will be excluded from study participation, if the patient withdraws his informed consent due to any reasons.
- for participating centers: Recruitment and data assessment of the follow-up examinations will be monitored by the Data Safety and Monitoring Board throughout the entire trial period. For recruitment of study patients, we have defined milestones at different time-points. If recruitment at the 50% landmark is below 20% of the total targeted number of patients, the enrolling center will have to be excluded from participating in the trial.
- for the whole trial: If recruitment is not achievable in more than one center, an interim analysis of the achieved effect size and subsequent reanalyses of the required sample size will reveal whether continuation of the trial is still realistic.

### Strategies to improve adherence to interventions {11c}

For the total duration of the trial, a telephone line will be opened up, and patients are encouraged to call any time in case that questions regarding the trial procedure occur. Further, to enhance confidentiality, relatives of all participating patients will have the possibility to take part in a training course on meditation and aromatherapy.

### Relevant concomitant care permitted or prohibited during the trial {11d}

This trial does not prohibit other treatments.

### Provisions for post-trial care {30}

After the completion of the interventions, follow-up visits will take place after 3 months, 6 months, and 12 months.

### Outcomes {12}

Primary and secondary outcomes will be assessed at baseline, after the 4-week intervention, and during follow-up visits at 3, 6, and 12 months.

#### Primary outcomes

Pain intensity: Assessed using the Numerical Rating Scale (NRS).

Perceived stress: Evaluated using the Perceived Stress Questionnaire (PSQ).

Quality of life: Measured using the EQ-5D questionnaire.

#### Secondary outcomes

Pain medication consumption: Recorded based on patient self-reporting and medical records.

Biomarkers of stress and inflammation:

Serum cortisol, C-reactive protein (CRP), and interleukin-6 (IL-6) will be measured at baseline and immediately after the 4-week intervention.

### Participant timeline {13}

**Fig. 2:**
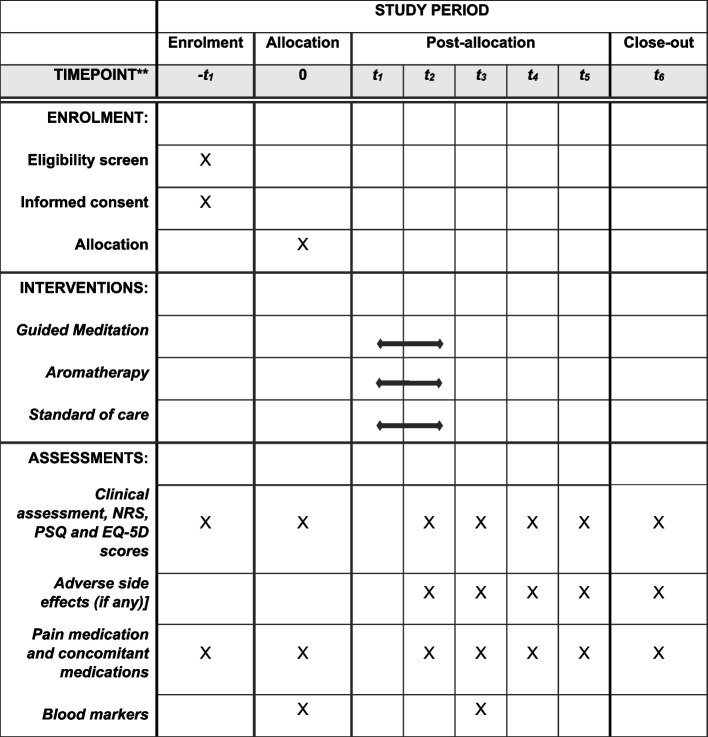
Schedule of enrollment, interventions, and assessments.* *t*_3_: 3 months after the intervention, *t*_4_: 6 months after the intervention, *t*_5_: 12 months after the intervention, *t*_6_: 24 months after the intervention

**Fig. 3:**
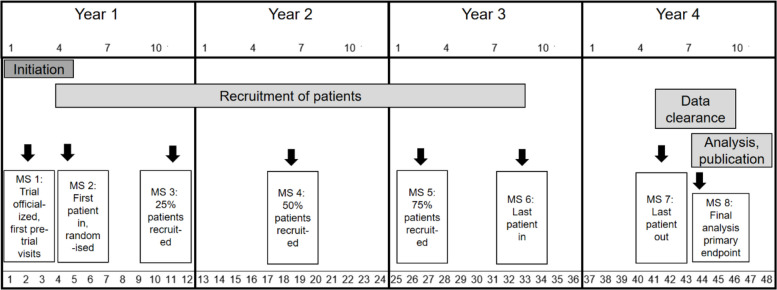
Trial timeline flow

The participant timeline is given in Fig. 2 The timeline of the whole trial is shown in Fig. 3.

### Sample size {14}

Assumptions: To determine the required sample size, we based our assumptions on previous studies investigating similar psychosomatic interventions in patients with chronic pain conditions. We assume the following for the primary outcome (pain reduction):

Effect size: A medium effect size of 0.5 is expected between the intervention groups and the control group.

Significance level (*α*): A two-sided significance level of 0.05 will be used.

Power (1-*β*): To detect a clinically meaningful difference, we aim for a statistical power of 80% (*β* = 0.2).

Drop-out rate: We anticipate a drop-out rate of 15% during the follow-up period.

Sample Size Calculation:

Using these assumptions, we calculated the required sample size using G*Power software for analysis of covariance (ANCOVA), with baseline pain scores as a covariate:

Minimum required participants per group: 30 participants in each group (guided meditation, aromatherapy, and control) are required to detect a medium effect size with 80% power at a significance level of 0.05.

Total sample size: Accounting for a 15% drop-out rate, the total number of participants to be recruited is 104 participants.

### Recruitment {15}

All patients admitted to the participating centers will be screened for potential recruitment. A daily 24-h report of all patients with musculoskeletal disorders will be extracted from the electronic database. Based on this extract, the written and electronic medical records of potential participants will be screened for eligibility by the study team. Eligible participants will be approached in person in the physical therapy practices. All of the information required for ensuring participant eligibility is obtained as part of routine care.

### Assignment of interventions: allocation

#### Sequence generation {16a}

Participants will be randomized in a 1:1:1 allocation ratio to guided meditation, aromatherapy, or standard care. The allocation sequence will be generated using a computer-based random number generator (www.randomizer.net) by an independent data management team not involved in recruitment or intervention delivery. Block randomization with randomly varying block sizes (e.g., 6 and 9) will be used to ensure balanced group allocation and prevent prediction of future assignments. Randomization will be stratified by study center to account for potential center effect.

#### Concealment mechanism {16b}

The allocation sequence will not be accessible to investigators, care providers, or outcome assessors. After confirmation of eligibility and completion of baseline assessments, study personnel will log into the secure platform to obtain the group assignment. The allocation will be revealed only after participant enrollment, thereby preventing selection bias.

#### Implementation {16c}

The allocation sequence will be created and maintained by the independent data management team at the University Hospital Regensburg. Participants will be enrolled by trained study personnel at each participating center. After obtaining written informed consent and completing baseline assessments, the enrolling investigator or study nurse will access the secure randomization platform and enter the participant’s study ID. The platform will automatically assign the participant to one of the three study arms and generate an electronic allocation record. The study monitor will have read-only access to the randomization log for auditing purposes.

### Assignment of interventions: blinding

#### Who will be blinded {17a}

The care providers and outcome assessors will be blinded using a single-masked procedure.

#### Procedure for unblinding if needed {17b}

No unblinding procedure is planned.

### Data collection and management

#### Plans for assessment and collection of outcomes {18a}

The primary and secondary outcomes will be assessed using validated and reliable instruments at baseline (pre-intervention), immediately after the 4-week intervention, and at follow-up visits 3, 6, and 12 months post-intervention.

#### Primary outcomes

Pain intensity: Pain intensity will be measured using the NRS, where participants rate their pain on a scale from 0 (no pain) to 10 (worst pain imaginable). The NRS is widely used in clinical settings and has demonstrated good reliability and validity for measuring pain in musculoskeletal conditions [22].

Perceived stress: Stress levels will be evaluated using the Perceived Stress Questionnaire (PSQ). The PSQ is a reliable self-report instrument commonly used in clinical research to assess subjective stress. It has been validated in various populations, including those with chronic pain [23].

Quality of life: Quality of life will be measured using the EQ-5D questionnaire, which assesses five dimensions of health: mobility, self-care, usual activities, pain/discomfort, and anxiety/depression. The EQ-5D is widely used and has been validated across diverse populations, making it a robust tool for assessing health-related quality of life [24].

#### Secondary outcomes

Pain medication consumption: This will be assessed by reviewing patient medical records and self-reported medication usage during follow-up visits. Participants will provide details of their pain medication use throughout the study period, including type, dosage, and frequency.

Biological sample collection: Venous blood samples will be collected at baseline and after completion of the 4-week intervention. Samples will be processed and stored at − 80 °C until batch analysis. Serum cortisol will be measured using immunoassay methods, and inflammatory biomarkers (CRP and IL-6) will be analyzed using standard ELISA techniques in an accredited laboratory.

Data collection process: Data will be collected by trained study nurses and investigators using standardized electronic case report forms (eCRF). To ensure data quality, all assessors will receive training on the use of study instruments and data collection procedures prior to the start of the trial.

Duplicate measurements will be taken for key variables (e.g., pain intensity) to ensure accuracy. Regular audits of the eCRF will be conducted by the study data management team to identify any missing or inconsistent data.

The Data Safety and Monitoring Board (DSMB) will periodically review the data to ensure ongoing trial integrity.

### Plans to promote participant retention and complete follow-up {18b}

The trial management will monitor all patient follow-up and will contact patients who missed a follow-up appointment. Also, the dates for follow-up visits are set at the end of the previous appointment and patients are reminded of the appointment one week before the scheduled appointment by phone or email. A 14-day window, defined as 7 days before and 7 days after the due date, will be available to complete the follow-up visits.

### Data management {19}

For study data collection, a web-based electronic case report form (eCRF) will be set up within an FDA 21 CFR Part 11 and ICH E6(R2) compliant clinical database management system (CDMS). All data management activities will comply with rules according to the EU-GDPR, including pseudonymized data storage.

Each investigator is responsible to review and ensure the accuracy, completeness, and timeliness of the data reported in the patient’s data entered in the eCRF and will provide his/her signature and date of signature on the eCRF pages. During the study, field monitors will review the eCRF entries by remote, and if necessary, by on-site source data verification in order to ensure accuracy, completeness, and plausibility of data entered. In addition, a central statistical monitoring approach will be applied to improve data quality and site performance. Data entered into the study database will be systematically and periodically checked by senior data management staff for completeness, for omissions, and values requiring further clarification using computerized and manual procedures. Any errors or omissions are entered on Data Query Forms, which are forwarded to the study site for resolution. Quality control audits of all key safety and efficacy data in the database are made prior to locking the database. After study completion, all electronic study data will be transferred to an auditable and system-independent accessible standard data format (CDISC) and stored for at least 10 years.

### Confidentiality {27}

Data will be collected pseudonymized and stored on a server at the University Hospital Regensburg with strictly controlled access for ensuring confidentiality. All analysis will be conducted with deidentified data.

### Plans for collection, laboratory evaluation and storage of biological specimens for genetic or molecular analysis in this trial/future use {33}

Blood samples will be collected for measurement of stress and inflammatory biomarkers (serum cortisol, CRP, IL-6). Samples will be stored in pseudonymized form in the institutional biobank for up to 10 years for analyses related to the study objectives.

## Statistical methods

### Statistical methods for primary and secondary outcomes {20a}

#### Primary estimand

The point estimates for both treatment arms will be presented as mean and standard deviation accompanied by the corresponding 97.5% confidence interval and will be compared between the meditation, the aromatherapy intervention and standard of care. To test the null hypothesis H_0_: μ_diff_ = 0 at a two-sided significance level of 0.025, an analysis of covariance (ANCOVA) with the respective component score at month 6 as dependent variable, treatment arm and center as fixed factor, and pain score at baseline, sex, and age as additional covariates will be used. Results will be presented using estimated marginal means of the difference between both groups accompanied by corresponding 97.5% confidence intervals. The study will be considered as successful if at least one component score shows a significant difference between both treatment arms. Two-sided alpha will be set at 0.025 to adjust for multiple testing.

#### The secondary estimand

Statistical analyses of the secondary endpoints will be carried out in an exploratory manner without any multiplicity adjustments. Descriptive safety analyses will be provided.

### Interim analyses {21b}

If recruitment is not achievable in more than one center, an interim analysis of the achieved effect size and subsequent reanalyses of the required sample size will be performed to evaluate whether continuation of the trial is realistic.

### Methods for additional analyses {20b}

Depending on the results of the primary endpoint, subgroup analyses based on age, sex, and diagnosis will be performed.

### Methods in analysis to handle protocol non-adherence and any statistical methods to handle missing data {20c}

We expect no more than 20% missing values regarding primary endpoints, which are considered to be missing completely at random (MCAR) or missing at random (MAR). To account for missing values regarding primary endpoints, multiple imputation using the Markov chain Monte Carlo (MCMC) method will be used [25]. A sensitivity analysis using an ANOVA approach on ranks will be used to explore the robustness of inference from the initial model.

### Plans to give access to the full protocol, participant level-data and statistical code {31c}

The statistical analysis plan will be made available as an amendment of the primary paper. Individual deidentified participant data (including data dictionaries) will be shared through Zenodo, a European open-access data repository. The statistical code will be made available to interested researchers upon a reasonable request for a period of five years.

### Oversight and monitoring

#### Composition of the coordinating center and trial steering committee {5d}

Each recruiting treatment center will be represented by a cooperating investigator, who is responsible for coordination, performance of recruitment, randomization, performance of patient-blinded interventions, and follow-up examinations at the referring center.

To ensure adherence to the intervention scheme and quality of the performance of each recruiting center, an independent Data Safety and Monitoring Board (DSMB) has been established, consisting of three experienced MDs and researchers in the field of orthopedics and physiotherapy, who are not involved in the conduct or design of the trial and are not part of any of the involved medical institutions. The Board’s responsibility will be monitoring and verifying the proper conduct of the study with respect to randomization, blinding, and the intervention performance using the mandatory documentation during monitoring visits at the centers.

### Composition of the data monitoring committee, its role and reporting structure {21a}

Quality assurance will consist of a combination of remote monitoring and on-site monitoring. Remote monitoring will be done by the data manager and will focus on data flow and accuracy in completing the eCRF. If performance is below a pre-defined quality threshold, additional on-site monitoring visits will be scheduled. On-site monitoring will be commissioned to the CRO multi-service monitoring which specializes in monitoring of non-commercial IITs since 2000. The monitors are qualified according to ICH-GCP and DIN ISO 14155 and adhere to the CRO’s SOPs MON 002, 003, 007, and 008.

On-site monitoring starts with a pre-trial visit of each center in order to ensure each center’s capability to comply with the study protocol and with the recruitment of the adequate number of patients. The findings of the pre-trial monitoring visit will be summarized in a report that will be forwarded to the PI. Monitoring will follow a risk-based approach, and the study is assessed as a low-risk trial. Thus, 100% source data verification focuses on informed consent, inclusion/exclusion criteria, the primary endpoints, randomization, and adverse and intercurrent events. All other aspects of the trial will be subjected to a 20% source data verification. In addition to the pre-trial visit, on-site monitoring is scheduled five times during the recruitment period with an interval of 6 months. After the milestone “last patient out” is achieved, one additional close-out visit will be planned.

### Adverse event reporting and harms {22}

The occurrence of adverse and intercurrent events will be coded using the Medical Dictionary for Regulatory Activities (MedDRA) terminology and documented in an eCRF throughout the follow-up period by each participating center. The safety reports will be forwarded to the independent Data Safety and Monitoring Board (DSMB).

### Frequency and plans for auditing trial conduct {23}

Besides the pre-trial monitoring visit, interim visits of study sites for the purpose of quality assurance and data monitoring will take place every six months. In addition, the PI will have weekly meetings with the research staff monitoring for any concerns. The DSMB will meet regularly biannually.

### Plans for communicating important protocol amendments to relevant parties {25}

Any protocol amendments require external approval from the Ethics Committee of the University Hospital Regensburg. Modifications will only be made with the authorisation of the study team as well as the DSMB. In case of any modifications, the written participant information and consent form will be updated and signed again by all participants.

#### Dissemination plans {31a}

The data collected during this study will be presented at international meetings and conferences. Data from this study, both positive and negative findings, will be published open-access in a peer-reviewed journal. The statistical analysis plan will be made available as an amendment of the primary paper. Individual deidentified participant data (including data dictionaries) will be shared through Zenodo, a European open access data repository. Data and documents will be made available to interested researchers upon a reasonable request for a period of five years. After data analysis, a symposium will be organized which will be open to the public. In the format of posters and talks with subsequent fishbowl discussions, detailed information regarding the state of the art in the diagnosis and treatment of musculoskeletal disorders will be provided in order to shape the direction of future research and outline possibilities to enhance subjective outcomes of patients.

## Discussion

The introduction of adjunct psychosomatic interventions in the treatment of musculoskeletal disorders represents a significant step toward integrating a more holistic model of care in clinical practice. Musculoskeletal conditions, such as chronic back pain and arthritis, are often treated with a narrow focus on physical symptoms, neglecting the psychological and emotional components that can exacerbate pain and negatively affect quality of life. This trial seeks to explore the potential benefits of integrating guided meditation and aromatherapy into conventional treatment pathways, addressing both physical and psychological factors that contribute to the burden of these disorders.

By adopting a biopsychosocial approach, this trial aligns with growing evidence that chronic pain is influenced by a combination of biological, psychological, and social factors. Such an approach recognizes that interventions targeting stress reduction, relaxation, and mental well-being can have a tangible impact on pain perception and recovery. If successful, the findings from this trial could reshape clinical guidelines by validating the use of psychosomatic therapies as complementary treatments for chronic musculoskeletal pain.

One of the key strengths of this trial is its multidisciplinary framework. By comparing guided meditation, aromatherapy, and standard care, this study offers a direct evaluation of how these complementary interventions might improve patient outcomes beyond what conventional care can achieve. This trial will also provide critical data on the acceptability of these interventions among patients, which is particularly important for treatments that rely on patient engagement and adherence.

However, recruitment and retention remain key challenges in a trial of this nature. While the proposed interventions are non-invasive and have the potential for high patient acceptability, some participants may find it difficult to adhere to the prescribed regimen of regular meditation or aromatherapy. The long-term follow-up period of 12 months further compounds this challenge, as participants might lose motivation or experience competing priorities over time. To address this, the trial incorporates strategies such as frequent follow-up reminders, personalized contact, and offering flexibility in scheduling, all of which are designed to improve retention and minimize attrition.

In terms of operational considerations, the trial is designed to be implemented across multiple centers, which introduces the complexity of maintaining consistency in the delivery of interventions and data collection. Ensuring that each center adheres to the same protocols is essential for minimizing variability and ensuring that results are comparable. This will require robust training for study staff and ongoing monitoring to verify compliance with the study procedures. The use of centralized randomization and standardized outcome measures will also help to mitigate potential inconsistencies across different sites.

Another potential challenge is related to the expected effect size of the interventions. Since few prior studies have explored the impact of psychosomatic therapies specifically for musculoskeletal conditions, there is some uncertainty regarding the magnitude of the expected effects. The sample size calculation is based on assumptions derived from studies in other therapeutic areas, which may not fully translate to this population. An interim analysis will be conducted to assess whether recruitment and effect sizes are meeting expectations, with adjustments to the trial design or sample size being considered if necessary.

Lastly, the design of the trial acknowledges the difficulties in blinding participants to psychosomatic interventions such as guided meditation and aromatherapy, which inherently depend on participant engagement. While this limitation could introduce bias, outcome assessors will be blinded to the allocation to reduce subjective influences during data collection. Furthermore, the use of objective, validated measures for pain, stress, and quality of life will ensure that the trial outcomes are as reliable as possible, despite the lack of full blinding.

This trial has several important strengths. First, the randomized controlled design and multicenter setting enhance internal and external validity. Second, the study evaluates two easily scalable, low-cost psychosomatic interventions that can be readily implemented in routine care. Third, the combination of validated patient-reported outcome measures with objective biological markers of stress and inflammation strengthens the scientific rigor and interdisciplinary relevance of the trial. The long-term follow-up period of 12 months further allows evaluation of sustained intervention effects.

Nevertheless, several limitations should be acknowledged. Blinding of participants to behavioral interventions is not feasible and may introduce expectancy effects. Adherence to home-based interventions may vary between participants despite strategies to support compliance. Additionally, the heterogeneous musculoskeletal patient population may increase variability in treatment response, although this reflects real-world clinical practice and enhances generalizability.

In summary, this trial is poised to provide valuable insights into the role of psychosomatic therapies as adjunct treatments for musculoskeletal disorders. By addressing both the psychological and physical dimensions of pain, the study has the potential to influence future clinical practice, promoting a more comprehensive and patient-centered approach to managing chronic musculoskeletal pain. The findings could also open the door for further research on the integration of complementary therapies into mainstream medical and physiotherapeutic treatments, ultimately improving patient outcomes and quality of life.

## Trial status

Latest protocol version 2, 15 October 2024, recruitment is scheduled by April 2025. Recruitment will be completed approximately in April 2027.

## Data Availability

Data will be stored on a server at the University Hospital Clinic Regensburg with strictly controlled access (researchers only) for ensuring confidentiality. The deidentified data collected during this study will be shared through Zenodo, a European open-access data repository.

## Abbreviations

DSMB: Data Safety and Monitoring Board
eCRF: Electronic case report form
MCMC: Markov chain Monte Carlo
MedDRA: Medical Dictionary for Regulatory Activities
